# Integrated Analysis of Cerebral Small Vessel Disease and Facial Soft-Tissue Markers in the Alzheimer’s Disease Continuum

**DOI:** 10.3390/brainsci16040403

**Published:** 2026-04-09

**Authors:** Caterina Bernetti, Gianfranco Di Gennaro, Roberta Roberti, Milena Ricci, Francesco Pipitone, Marta Profilo, Francesco Motolese, Rosalinda Calandrelli, Fabio Pilato, Vincenzo Di Lazzaro, Bruno Beomonte Zobel, Carlo Augusto Mallio

**Affiliations:** 1 Fondazione Policlinico Universitario Campus Bio-Medico, 00128 Rome, Italy; 2 Research Unit of Diagnostic Imaging, Department of Medicine and Surgery, Università Campus Bio-Medico di Roma, 00128 Rome, Italy; 3 CRUISE Research Center, Science of Health Department, Magna Græcia University, 88100 Catanzaro, Italy; 4 Research Unit of Neurology, Department of Medicine and Surgery, Università Campus Bio-Medico di Roma, 00128 Rome, Italy; 5 Radiology and Neuroradiology Unit, Department of Imaging, Radiation Therapy and Hematology, Università Cattolica del Sacro Cuore, Fondazione Policlinico Universitario Agostino Gemelli—IRCCS, 00168 Roma, Italy

**Keywords:** Alzheimer’s disease (AD), mild cognitive impairment (MCI), cerebral small vessel disease (CSVD), sarcopenia, masseter muscle volume, perivascular spaces (PVSs), magnetic resonance imaging (MRI), biomarker

## Abstract

**Highlights:**

**What are the main findings?**

- The study identified a significant multivariate correlation (r = 0.51) between high Cerebral Small Vessel Disease (CSVD) burden and a worse facial soft-tissue profile, characterized by muscle atrophy and fat infiltration.
- Masseter muscle volume was significantly reduced in Alzheimer’s Disease (AD) patients compared to those with Mild Cognitive Impairment (MCI), while perivascular spaces in the midbrain emerged as the strongest neuroradiological predictor for AD.

**What are the implications of the main findings?**

- Quantitative facial soft-tissue metrics, such as masseter volume and quality, can serve as non-invasive peripheral biomarkers for systemic frailty and neurodegeneration within the AD continuum.
- These findings support the “muscle–brain axis” hypothesis, suggesting that sarcopenia and cerebral vascular pathology are interconnected manifestations of a shared pathobiological process.

**Abstract:**

**Objective:** To investigate the integrated relationship between Cerebral Small Vessel Disease (CSVD) markers and quantitative facial soft-tissue measurements in Alzheimer’s disease (AD) continuum, utilizing peripheral muscle health as a potential biomarker for systemic frailty and neurodegeneration. **Methods:** Retrospective analysis of 3T brain MRI data from 67 patients (AD, N = 45; Mild Cognitive Impairment [MCI], N = 22). CSVD markers were assessed using STRIVE and standardized scales (Fazekas, Potter). Facial soft-tissue metrics, including masseter and tongue volume, temporal muscle thickness (TMT), and fat infiltration (Mercuri Scale), were quantified via semi-automatic segmentation on T1-weighted sequences. Group comparisons (AD vs. MCI) used regression models adjusted for age and sex. The overall central–peripheral relationship was explored via Canonical Correlation Analysis (CCA). **Results:** The AD group showed a highly significant cognitive decline (MMSE: 23.2 ± 4.1 vs. 28.2 ± 1.4, *p* < 0.0001). Centrally, the presence of PVSs in the mesencephalic region was the most robust predictor for AD (*p* = 0.003). Peripherally, average masseter muscle volume was significantly lower in the AD group (*p* = 0.0273), and masseter fat infiltration was significantly higher (*p* = 0.025), supporting localized sarcopenia. The CCA demonstrated a statistically significant positive multivariate relationship (r = 0.51, Roy’s Largest Root *p* = 0.015) between a higher combined CSVD burden and a worse soft tissue profile across the cohort. **Conclusions:** Quantitative indices of facial soft tissues, particularly masseter muscle volume and quality, reflect systemic frailty and cognitive deterioration along the AD continuum. The strong central–peripheral correlation suggests that sarcopenia and CSVD are interconnected manifestations of a shared pathobiological process. These easily measurable facial markers could serve as valuable, non-invasive peripheral biomarkers, complementing traditional neuroimaging risk stratification in AD.

## 1. Introduction

Alzheimer’s Disease (AD) is the most prevalent form of neurodegenerative dementia, characterized by a progressive decline in cognitive functions. Globally, the burden of dementia is substantial, with an estimated 55 million cases in 2020, projected to rise to 139 million by 2050 [1]. The diagnostic framework has evolved from purely clinical to a biomarker-based approach, utilizing the beta-amyloid (Aβ), Tau, and neurodegeneration (ATN) classification, recognizing AD as a continuum starting with preclinical changes, such as Mild Cognitive Impairment (MCI) [2]. Magnetic Resonance Imaging (MRI) is a cornerstone in diagnosis, particularly for identifying structural markers such as atrophy and assessing the overall burden of Cerebral Small Vessel Disease (CSVD) [2,3].

CSVD, which encompasses white matter hyperintensities (WMHs), perivascular spaces (PVSs), lacunes, and cerebral microbleeds (CMBs), shares common vascular risk factors with AD and often coexists with neurodegenerative pathology, accelerating cognitive decline [4,5]. Notably, the presence of PVSs suggests a dysfunction of the glymphatic system, impacting the clearance of metabolic waste, including Aβ [6,7].

Furthermore, emerging evidence suggests a strong link between AD and systemic frailty, encapsulated by the concept of sarcopenia—an age-related muscle condition defined by reduced muscle strength and mass/quality [8,9]. This association is often conceptualized through the “muscle–brain axis,” a bidirectional dialogue where myokines and neurotrophic factors link muscle health to cognitive reserve [8,10,11]. Quantitative MRI of the facial soft tissues, particularly the temporal and masseter muscles, is an effective and non-invasive method for assessing localized sarcopenia, correlating with systemic frailty and adverse clinical outcomes in neurological cohorts [12,13,14].

The general objective of this study is to explore the relationships between brain radiological features (CSVD markers) and quantitative morphometric measures of facial soft tissues in individuals with confirmed AD or MCI [13,14,15,16,17]. The first aim is to compare two groups of patients with respect to a set of cerebral radiological variables and a set of facial morphological variables. Furthermore, it seeks to evaluate the associations between the brain radiological alterations and the facial soft-tissue characteristics, in order to determine whether and to what extent structural changes in the brain are reflected in subcutaneous components of the face. A further objective is to investigate whether the relationship between central and peripheral structures differs between the different stages of the AD spectrum, under the hypothesis that the progression of neurodegeneration may modify this link. Finally, the study examines the relationship between structural variables and cognitive performance, as assessed by the Mini Mental State Examination (MMSE), as an indicator of cognitive impairment [18]. Overall, the study aims to contribute to an integrated understanding of Alzheimer’s Disease, considering not only the brain but also subcutaneous facial tissues, within an extended anatomo-functional framework.

By comparing these two clinical stages and performing a multivariate correlation analysis, we sought to determine if some facial features, used as indicators of sarcopenia/frailty and easily extractable in a normal brain MRI, could be utilized as readily measurable biomarkers alongside traditional neurodegeneration and CSVD indices in the Alzheimer’s continuum.

## 2. Materials and Methods

### 2.1. Study Design, Population

The study was approved by the local Ethics Committee and conducted in accordance with the principles of the Declaration of Helsinki.

Sixty-seven patients were enrolled by the Neurology Unit of Fondazione Policlinico Universitario Campus Bio-Medico di Roma, following suspected neurodegenerative disease and all undergoing 3T brain MRI between September 2024 and June 2025. Data from the electronic medical records of the patients included were retrospectively analyzed.

Inclusion criteria were: (1) patients referred for evaluation of AD or MCI with a complete MMSE test; (2) the presence of high-quality 3T structural MRI with a complete protocol; (3) patients older than 18 years.

Exclusion criteria were: (1) the presence of fixed dental prostheses causing ferromagnetic artifacts with severe reduction in image quality; and (2) failure to complete the entire planned brain MRI protocol.

The following variables were collected for each patient: age, gender, MMSE results and confirmed diagnosis of AD or MCI.

### 2.2. Image Acquisition and Analysis

**Scanning Parameters**

Images were acquired using a 3T MRI system (Lumina; Siemens Healtineers, Erlangen, Germany), configured with a 32-channel head coil, at the Diagnostic Imaging Unit of Fondazione Policlinico Universitario Campus Bio-Medico di Roma.

The specific protocol included detailed parameters provided in Table 1.

### 2.3. Image Analysis

The MRI images were evaluated in the DICOM (Digital Imaging and Communications in Medicine) format using the PACS system on the same workstation.

**Figure 1:**
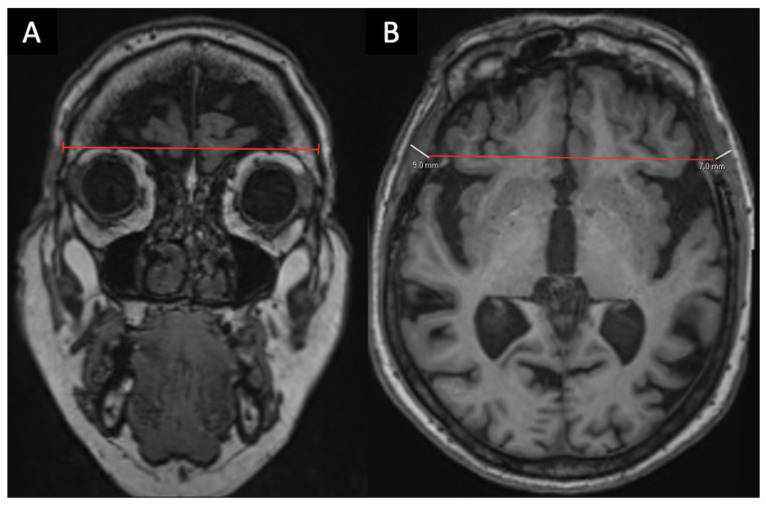
**Measurement of Temporal Muscle Thickness (TMT).** In order to ensure reproducibility, measurements of TMT (mm) are performed on axial T1-weighted sequences in the axial plane (**B**) using a simple measurement tool on the PACS system, as the maximum perpendicular distance between the outer table of the cranium and the inner layer of the superficial temporal fascia, typically at the level of the superior orbital roof, found in the coronal plane (**A**).

**Figure 2:**
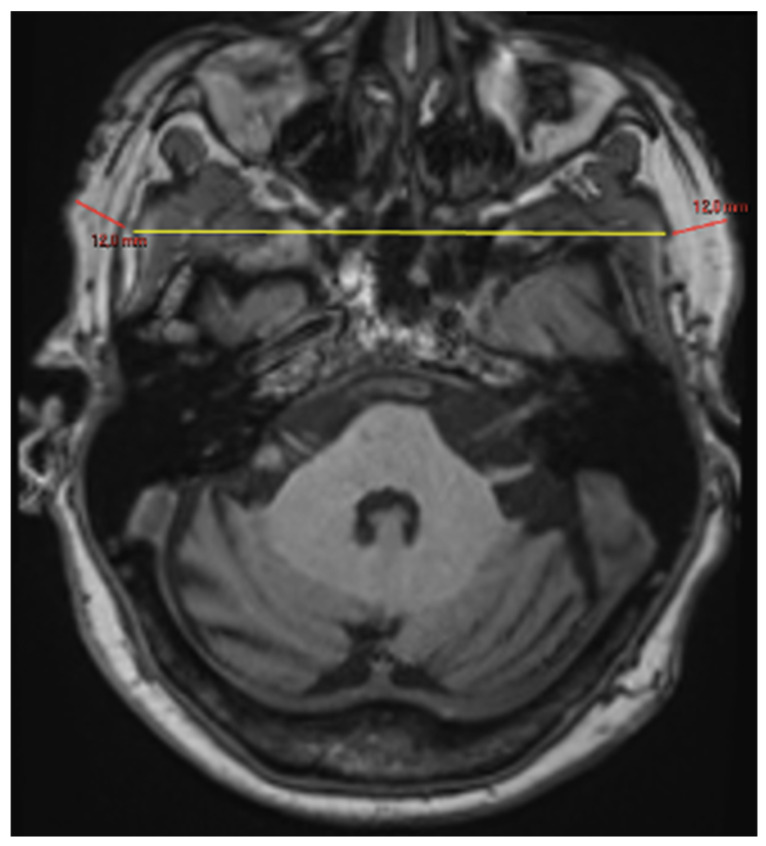
**Measurement of Subcutaneous Fat Thickness (SFT).** In order to ensure reproducibility, measurements of SFT (mm) are performed on axial T1-weighted sequences in the axial plane using a simple measurement tool on the PACS system, as the maximum perpendicular distance between the fascia and skin, found in the axial plane.

The following quantitative metrics were measured (Table 2):

- **Thickness measurements** (mm):
  ○ Temporal Muscle Thickness (TMT), right, left and average, in axial views [13,17,19] (Figure 1);
  ○ Subcutaneous Fat Thickness (SFT), in axial views (Figure 2);

Thickness was evaluated on T1-weighted sequences using a simple linear measurement tool on the PACS system.

**Figure 3:**
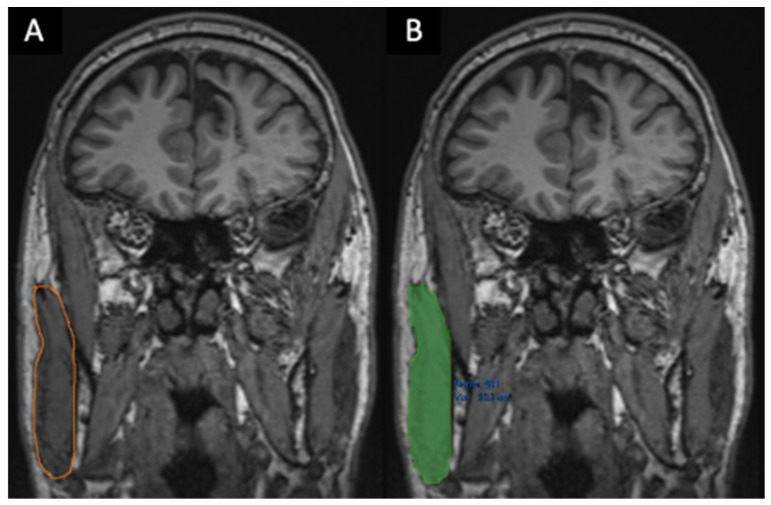
**Volume quantification of masseter muscles** (**A**,**B**). Volumes were quantified in cubic centimeters (cm^3^) using semi-automatic segmentation (Vue PACS “Livewire Mode”) on T1-MPRAGE sequences. Delineation was primarily performed on coronal planes, utilizing axial and sagittal views as anatomical references for complete tridimensional volume calculation.

**Figure 4:**
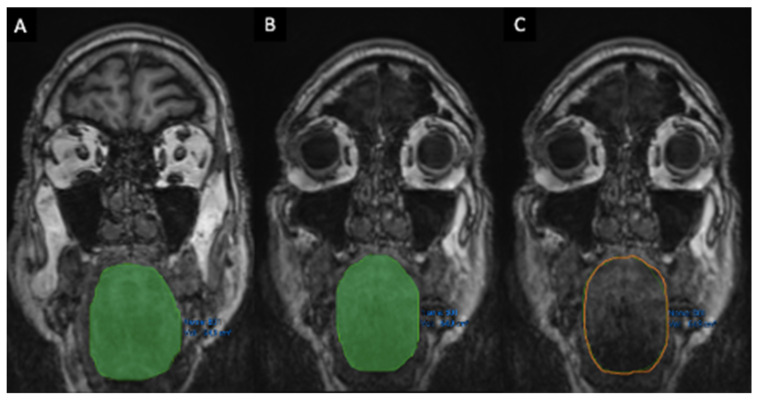
**Volume quantification and qualitative assessment of tongue muscles** (**A**–**C**). Volumes were quantified in cubic centimeters (cm^3^) using semi-automatic segmentation (Vue PACS “Livewire Mode”) on T1-MPRAGE sequences. Delineation was primarily performed on coronal planes (**A**–**C**), utilizing axial and sagittal views as anatomical references for complete tridimensional volume calculation.

- **Volume measurements** (cm^3^):
  ○ Masseter muscle volume (MMV), right, left and average, in coronal views [14,15] (Figure 3);
  ○ Tongue volume (TV), in coronal views (Figure 4) [14,16].

Segmentations were performed on **T1-weighted sequences** using the Vue PACS software (v11.14) via the “Livewire mode Segmentation” function, which provides a semi-automatic segmentation method that also allows for the manipulation of lesion contours.

**Figure 5:**
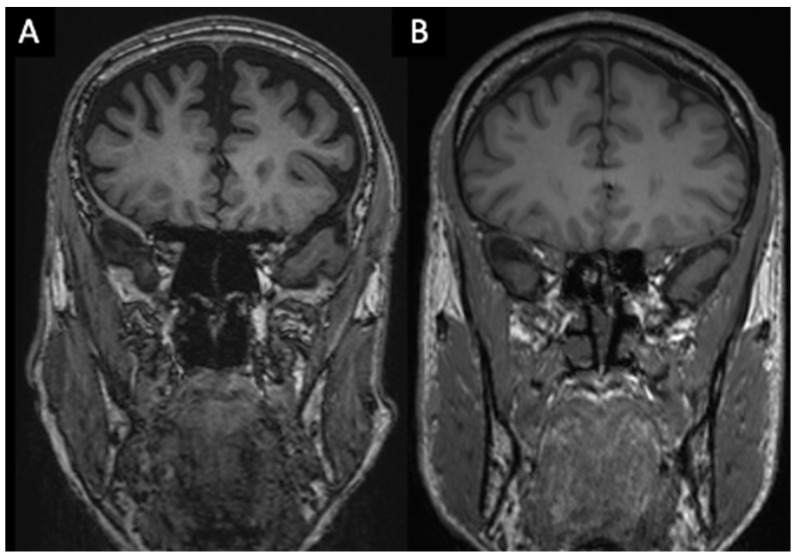
**Qualitative assessment, intended as fat infiltration (FI) of tongue muscles.** FI was visually graded using the Mercuri Scale (0–4), a qualitative rating system for miosteatosis. Images show grade 1 (**A**) and grade 4 (**B**) with respect to FI.

- **Fat Infiltration—FI** (Mercuri Scale) [20,21,22]:
  ○ Masseter muscle FI;
  ○ Tongue FI (Figure 5).

FI was graded from 0 to 4—from less to more severe—using the Mercuri Scale, a qualitative visual rating system used to assess the severity of miosteatosis (or FI) within a muscle [23,24].

A suitably trained operator evaluated and segmented all images.

### 2.4. Assessment of CSVD Markers

A comprehensive assessment of CSVD-related imaging markers was performed in accordance with the STRIVE (STandards for Reporting Vascular changes on nEuroimaging) criteria. The evaluation included the identification and quantification of all recognized CSVD features: cerebral microbleeds (CMBs), Cortical Superficial Siderosis, Recent Subcortical Infarcts, Cortical Microinfarcts, lacunes, atrophy, WMHs and PVSs. Specific quantitative and qualitative grading scales were employed: the Fazekas scale was used for rating WMH burden; PVSs were quantified using the Potter criteria, employing an ordinal scale for basal ganglia (0–4) and centrum semiovale (0–4); while a binary assessment (1: presence vs. 0: absence) was used for the midbrain (MB). All CSVD markers were evaluated on the appropriate T1-MPRAGE, T2-FLAIR, and T2*-SWI sequences by a suitably trained operator.

### 2.5. Statistical Analysis

All statistical analyses were performed using appropriate parametric or non-parametric methods, depending on the type and distribution of the variables. Categorical and binary variables were summarized as absolute frequencies and percentages and compared between Alzheimer and MCI groups using the chi-square or Fisher’s exact test, as appropriate. Continuous variables were summarized using minimum, maximum, median, interquartile range, mean, and standard deviation. The normality of distributions was assessed by the Shapiro–Wilk test. Depending on the result of this test, comparisons between groups were conducted using the independent samples *t*-test or the Mann–Whitney U test.

For each variable of interest, additional regression models were fitted to adjust for potential confounding by age and sex. Binary outcomes were analyzed using logistic regression, ordered outcomes were examined through ordered logistic regression, and continuous outcomes were analyzed using linear regression, with group (Alzheimer vs. MCI) as the main independent variable. Standard diagnostic procedures were performed for all regression models to ensure valid estimations.

To explore the relationships between radiological and facial soft-tissue variables, a Canonical Correlation Analysis (CCA) was conducted separately within the Alzheimer and MCI groups. Specifically, individual CSVD markers (lacunes, Fazekas scale, perivascular spaces, microbleeds, cortical siderosis, and atrophy) were included as one variable set and facial soft-tissue measures as the second set. This multivariate technique was used to identify pairs of canonical functions representing linear combinations of variables that maximize the correlation between the two sets. Prior to the analysis, multicollinearity within each set was assessed, and redundant variables were excluded to ensure model stability and interpretability. The canonical loadings, canonical correlations, and percentage of variance explained by each canonical function were examined to characterize the strength and structure of the associations. To assess the precision of the first canonical correlation, a bootstrap procedure with 1000 replications was performed, and a 95% confidence interval was estimated.

All statistical tests were two-tailed, and a *p* value < 0.05 was considered statistically significant.

## 3. Results

### 3.1. Demographic and Clinical Characteristics

The study included 67 patients for comparative analysis, divided into an AD group (N = 45) and an MCI group (N = 22). The overall mean age of the sample was 75.6 ± 8.2 years, with no statistically significant differences in age between the two groups (AD: 75.3 ± 8.8 years vs. MCI 76.4 ± 7.1 years; *p* = 0.187). The sample was predominantly female (71.6), although the raw analysis did not show significant differences in sex distribution between the groups. Clinically, the MMSE score confirmed significant cognitive deterioration in the AD group (23.2 ± 4.1) compared to the MCI group (28.2 ± 1.4), with a highly significant difference in the adjusted regression model (*p* < 0.0001). This clinical dissociation, in the absence of a significant age difference between the groups, validates the comparison for the identification of specific radiological and morphometric biomarkers.

### 3.2. Analysis of Clinical and Imaging Findings (Figure 3 and Figure 4)

The presence of PVSs in the MB was significantly associated with the AD group (*p* = 0.003). Bivariate analysis did not reveal a significant association between the presence of CMBs and the diagnosis of AD (*p* = 0.363). However, in the logistic regression model adjusted for age and sex, CMBs were a statistically significant predictor, though with an inverse correlation (OR = 0.229, 95% CI: 0.054, 0.971; *p* = 0.046).

Linear regression analysis confirmed the association between muscle atrophy and the AD group. Specifically, the volume of the right masseter was significantly lower in the AD group (*p* = 0.023). While the volume of the left masseter did not reach the significance threshold, its value was borderline (*p* = 0.052), suggesting a trend toward atrophy that nonetheless supports the bilateral finding, albeit with a right-sided prevalence in this sample. In conclusion, a significant reduction in the average masseter muscle volume was found overall in the AD group (19.47 cm^3^) compared to the MCI group (22.68 cm^3^) (*p* = 0.027). Furthermore, regarding this muscle, ordered logistic regression analysis also revealed that adipose infiltration (miosteatosis) of the masseter, according to the Mercuri Scale, is significantly more probable in the AD group (*p* = 0.025). The thickness of the right temporal muscle also did not reach the significance threshold but was a borderline value (*p* = 0.052) (Table 3 and Table 4).

### 3.3. Canonical Correlation Analysis

A Canonical Correlation Analysis (CCA) was performed on the entire cohort (N = 67) to assess the overall multivariate relationship between a set of radiological markers of CSVD and a set of soft-tissue characteristics. To ensure consistent clinical interpretation across all variables, the scales of Temporal Muscle Thickness, masseter volume, and tongue volume were inverted so that higher values indicate worse outcomes for all measures, aligning with the directionality of the radiological markers.

**Figure 6:**
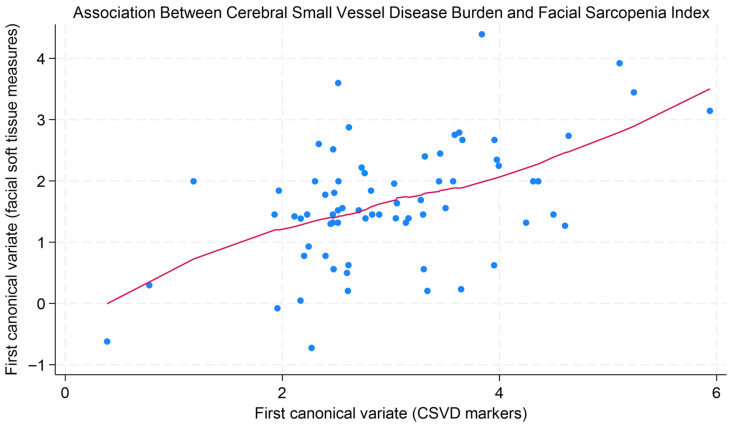
Relationship between multivariate representations of CSVD markers and facial soft-tissue measures. Each axis represents the first canonical variate derived from the two variable sets. The red line shows a LOWESS-smoothed trend.

The first canonical correlation was 0.51 (bootstrapped 95% CI: 0.39–0.63), indicating a moderate multivariate association. The relationship between the first pair of canonical variates was positive and is illustrated in Figure 6 using a LOWESS plot, which shows a generally increasing trend.

After scale harmonization, this pattern suggests that a higher combined burden of radiological markers tends to be associated with less favorable soft-tissue characteristics, including increased fat infiltration and reduced muscle volume. However, given the variability of the data and the overall pattern of results, this finding should be interpreted as exploratory.

The omnibus test for the set of all canonical correlations was not statistically significant (Wilks’ Lambda, *p* = 0.72). However, the test for the first and strongest canonical correlation was significant (Roy’s Largest Root, *p* = 0.015). This pattern, where a significant first correlation exists alongside a non-significant omnibus test, suggests limited statistical power. The sample size of 67 is modest for the high-dimensional CCA model, which estimates 48 parameters, potentially leading to reduced power to detect the full set of correlations.

## 4. Discussion

The findings of this study provide integrated evidence supporting the hypothesis that both MCI and AD are associated with quantifiable structural changes in the facial soft tissues, beyond traditional cerebral alterations, such as those that are CSVD related.

While the identified multivariate correlation is moderate, these findings suggest that facial markers could serve as exploratory, non-invasive peripheral biomarkers, complementing traditional neuroimaging in AD risk stratification.

Clinically, the most obvious finding reinforces the concept of a pathophysiological continuum linking central cognitive decline in patients in the AD continuum. In fact, our analysis confirmed the expected significant cognitive impairment in the AD group compared to the MCI group (*p* = 0.000), in accordance with the extensive literature using the MMSE as a cardinal marker for dementia progression [18].

The most robust neuroradiological predictor for the AD diagnosis was the significantly higher burden of PVSs in the MB (OR = 6.89, *p* = 0.003) in AD patients. This finding aligns with literature emphasizing the crucial role of glymphatic system dysfunction and CSVD in the progression of neurodegeneration, suggesting that the difficulty in clearing waste products is intensified in more advanced stages of the disease, which could also be determined by the presence of Aβ [4,6,7].

Conversely, CMBs were more diffused in the MCI group (OR = 0.229, *p* = 0.046). Even though this result stands in contrast with the typically reported association between AD and CMBs linked to Cerebral Amyloid Angiopathy (CAA) and hypertensive arteriopathy, this unexpected discrepancy warrants cautious interpretation and further evaluation. This dissociation underscores the importance of distinguishing pure vascular mechanisms, primarily driven by age and non-specific to AD, from those more strictly linked to neurodegeneration (such as PVSs). It also raises the hypothesis that the MCI group is clinically heterogeneous, potentially representing the initial stages of other pathological conditions that, despite sharing some characteristics like SVD, follow evolutionary pathways not always tied to AD.

The most compelling aspect of our work lies in the CCA, which established a significant multivariate association (r = 0.51, *p* = 0.015) between the global cerebral radiological burden and the unfavorable facial soft-tissue profile. This link supports the concept of a dysfunctional Brain–Muscle Axis in AD, suggesting that the processes of CSVD, neurodegeneration, and sarcopenia may be coexisting or interconnected manifestations along a single pathobiological continuum [8,9,11,13,17].

In fact, a higher combined cerebral burden correlated with a worse soft-tissue profile, particularly with increased adipose infiltration and reduced muscle volume.

Specifically, the masseter muscle emerged as the most sensitive soft-tissue marker. AD patients displayed a significantly reduced average MMV (*p* = 0.027), a difference that persisted after adjusting for age and sex. Furthermore, the masseter exhibited significant qualitative deterioration, with a strongly increased odds ratio for adipose infiltration in the AD group (OR = 5.104, *p* = 0.025), serving as a quantifiable indicator of systemic frailty. These changes in a crucial masticatory muscle reinforce the link hypothesized between sarcopenia, even in specific muscle compartments, and AD [8,9,10,11,17]. It is also noteworthy that the significance was more robust in the right masseter (*p* = 0.023), while the left was borderline significant (*p* = 0.052), providing support for a bilateral finding, albeit with asymmetry. This observed right-sided asymmetry in masseter atrophy is notable and warrants future investigation to assess the influence of mastication dominance, possibly related to handedness or dental status, which were not available for the current analysis.

Collectively, the findings underscore the utility of an extended anatomo-functional framework, where markers of cerebral microangiopathy and facial sarcopenia markers serve as integrated indicators of AD progression.

This study has several important limitations. First, the relatively small sample size, particularly in relation to the number of variables included in multivariate analyses, may increase the risk of overfitting and limit statistical power. For this reason, the Canonical Correlation Analysis should be interpreted as exploratory and hypothesis-generating.

Second, adjustment for confounding was limited to age and sex, as detailed information on vascular risk factors, nutritional status, BMI, dentition, and behavioral variables was not consistently available. Therefore, the observed associations should not be interpreted as causal and may reflect broader processes such as systemic frailty.

Third, no formal assessment of inter- or intra-rater reliability was performed, and all measurements were conducted by a single operator, potentially introducing measurement bias.

Fourth, multiple comparisons were performed without formal correction, increasing the risk of false positive findings, particularly for borderline results. In addition, some regression estimates may be unstable due to low counts in specific categories and should be interpreted with caution.

Fifth, cognitive characterization relied primarily on MMSE, which provides a global screening measure and does not capture domain-specific cognitive impairment.

Sixth, the cross-sectional design precludes any temporal or causal inference, and some observed associations, particularly those involving muscle measures, may reflect secondary consequences of disease progression (e.g., nutritional or behavioral changes) rather than primary disease mechanisms.

Finally, the absence of a healthy control group limits external validity, and the feasibility of the proposed imaging-based measurements remains currently confined to research settings, requiring further validation for clinical implementation.

## 5. Conclusions

In conclusion, this study demonstrates that quantitative indices of facial soft tissues, particularly masseter muscle volume and quality, correlate with cognitive deterioration along the AD continuum. These findings suggest that easily measurable facial features on routine brain MRI could represent potential, non-invasive peripheral indicators of systemic frailty, providing exploratory data that complement traditional neuroimaging markers of small vessel disease. Future large-scale prospective studies are essential to validate the prognostic value of these integrated biomarkers for risk stratification and longitudinal monitoring in clinical practice.

## Figures and Tables

**Table 1:** **MRI 3T brain acquisition protocol parameters.** This table lists the essential sequence parameters used for structural brain imaging on the 3 Tesla scanner. The protocol was specifically designed to evaluate both CSVD markers and facial soft tissues. (Abbreviations: CSVD, Cerebral Small Vessel Disease; FOV, Field of View; FS, Fat Saturation; MPRAGE, Magnetization Prepared Rapid Gradient Echo; SPACE, Sampling Perfection with Application optimized Contrasts using different flip angle Evolutions; SWI, Susceptibility Weighted Imaging; TE, Echo Time; TI, Inversion Time; TR, Repetition Time; TSE, Turbo Spin Echo).

Sequence	Orientation	TR (ms)	TE (ms)	TI (ms)	Matrix	FOV (mm)	Slice Thickness (mm)
T2 SPACE FLAIR	3D/Sagittal	5000	419	1640	256	282	0.5
RESOLVE 3SCAN TRACE	Axial	2910	62	-	160	220	3.0
T2 TSE FS	Coronal	4300	101	-	384	220	3.0
T2 TSE	Axial	4690	109	-	368	230	3.0
T1 MPRAGE	Sagittal	2100	3.37	-	256	256	1.0
SWI	Axial	27	20	-	224	230	2.0

**Table 2:** **Image analysis of facial soft tissues.** This table summarizes the specific measurements, planes of acquisition, software, and techniques used to quantitatively assess muscle volume, thickness, and fat infiltration (sarcopenia markers) in the facial region using 3T MRI. (Abbreviations: cm^3^, cubic centimeters; FI, fat infiltration; mm, millimeters; MMV, masseter muscle volume; MPRAGE, Magnetization Prepared Rapid Gradient Echo; PACS, Picture Archiving and Communication System; TMT, Temporal Muscle Thickness).

Measurement (Unit)	Variable Measured	Measurement Plane (s)	Software and Technique	Sequence
Thickness (mm)	Subcutaneous Fat Thickness	Axial	Simple linear measurement tool on the PACS system	T1-weighted MPRAGE
	TMT: Right, Left, and Average			
Volume (cm^3^)	MMV: Right, Left, and Average	Coronal for delineation Axial and sagittal as reference	Vue PACs (v11.14) “Livewire mode Segmentation” (semi-automatic)	
	Tongue Volume			
FI (Mercuri Scale)	Masseter	Coronal	Visual rating system	
	Tongue			

**Table 3:** **Comparison of continuous morphometric and cognitive variables between MCI and AD groups.** Descriptive statistics for each group were chosen based on the Shapiro–Wilk normality test. If the variable distribution was not significantly non-normal (*p* > 0.05), data are presented as mean (SD) and the unadjusted comparison uses the *t*-test (T). If the distribution was non-normal (*p* < 0.05), the median [IQR] and unadjusted comparison uses the Wilcoxon Rank-Sum test (W). The adjusted *p*-value is derived from a linear regression model with the AD group as the main predictor, adjusted for age and sex. Significant *p*-values (*p* < 0.05) are highlighted in gray; almost significant *p*-values are highlighted in lighter gray. Abbreviations: MMSE, Mini Mental State Examination.

Variables	MCI (N = 22)	Alzheimer (N = 45)	*p*	*p* (Adjusted)
MMSE	28.00 [28.00–29.00]	23.00 [21.00–27.00]	0.0000 (W)	0.000
MV Average (cm^3^)	22.65 [18.25–27.10]	18.45 [17.10–22.15]	0.0267 (W)	0.027
MV Right (cm^3^)	23.49 (6.15)	20.17 (5.30)	0.0259 (T)	0.023
MV Left (cm^3^)	21.20 [16.50–26.00]	18.10 [16.80–21.00]	0.0826 (W)	0.052
TV (cm^3^)	71.00 [59.90–77.80]	68.70 [59.90–75.10]	0.7235 (W)	0.970
TMT Average (mm)	8.95 (1.87)	8.31 (1.48)	0.1344 (T)	0.302
TMT Right (mm)	9.00 [7.00–10.40]	8.50 [7.70–9.20]	0.3761 (W)	0.052
TMT Left (mm)	8.90 (1.93)	8.10 (1.63)	0.0795 (T)	0.197
SFT (mm)	15.75 (2.41)	15.83 (2.18)	0.8935 (T)	0.576

**Table 4:** **Descriptive comparison of categorical and ordinal measures of CSVD and facial soft-tissue FI between MCI and AD groups.** The table reports the absolute counts (n) and percentages (%) of patients for each score level within the MCI and AD groups. Binary variables (lacunes, CMBs, cortical siderosis, and PVSs in the MB) were scored 0 (Absence) or 1 (Presence). WMHs were graded using the Fazekas scale (range 0–3). PVSs were assessed using ordinal scales specific to their location: BG (0–3) and SC (0–4). Masseter and lingual muscle FI were graded using the Mercuri Scale, (0–3). Group comparisons were performed using Fisher’s exact test (unadjusted *p*-value) and ordered logistic regression (adjusted OR and *p*-value), adjusted for age and sex.

Variable (Scale)		MCI (N = 22)	Alzheimer (N = 45)	*p*	OR (95% CI)	Adjusted *p*
CMBs (0, 1)	0	15 (68.2%)	36 (80.0%)	0.287 (chi2)	0.229 (0.054, 0.971)	0.046
	1	7 (31.8%)	9 (20.0%)			
Cortical Siderosis (0, 1)	0	19 (86.4%)	34 (75.6%)	0.246 (Fisher’s exact)	1.680 (0.388, 7.287)	0.488
	1	3 (13.6%)	11 (24.4%)			
Lacunes (0, 1)	0	19 (86.4%)	43 (95.6%)	0.321 (Fisher’s exact)	0.145 (0.015, 1.437)	0.099
	1	3 (13.6%)	2 (4.4%)			
PVSs MB (0, 1)	0	16 (72.7%)	15 (33.3%)	0.004 (Fisher’s exact)	6.886 (1.971, 24.060)	0.003
	1	6 (27.3%)	30 (66.7%)			
PVSs BG (0–4)	0	0 (0.0%)	0 (0.0%)	0.107 (Fisher’s exact)	2.695 (0.642, 11.309)	0.176
	1	19 (86.4%)	29 (64.4%)			
	2	2 (9.1%)	14 (31.1%)			
	3	1 (4.5%)	2 (4.4%)			
	4	0 (0.0%)	0 (0.0%)			
PVSs SC (0–4)	0	0 (0.0%)	0 (0.0%)	0.409 (Fisher’s exact)	1.301 (0.479, 3.529)	0.606
	1	5 (22.7%)	8 (17.8%)			
	2	13 (59.1%)	20 (44.4%)			
	3	3 (13.6%)	9 (20.0%)			
	4	1 (4.5%)	8 (17.8%)			
WMHs (0–3)	0	0 (0.0%)	2 (4.4%)	0.601 (Fisher’s exact)	0.521 (0.149, 1.815)	0.306
	1	17 (77.3%)	29 (64.4%)			
	2	3 (13.6%)	11 (24.4%)			
	3	2 (9.1%)	3 (6.7%)			
Masseter FI (0–3)	0	3 (13.6%)	1 (2.2%)	0.062 (Fisher’s exact)	5.104 (1.223, 21.292)	0.025
	1	17 (77.3%)	30 (66.7%)			
	2	2 (9.1%)	13 (28.9%)			
	3	0 (0.0%)	1 (2.2%)			
Tongue FI (0–3)	0	0 (0.0%)	0 (0.0%)	0.304 (Fisher’s exact)	3.098 (0.729, 13.164)	0.126
	1	19 (86.4%)	31 (68.9%)			
	2	3 (13.6%)	12 (26.7%)			
	3	0 (0.0%)	2 (4.4%)			

Abbreviations: AD: Alzheimer’s Disease; MCI: Mild Cognitive Impairment; PVSs: perivascular spaces; WMHs: white matter hyperintensities; OR: odds ratio.

## Data Availability

The data presented in this study are available on request from the corresponding author due to privacy.

## Abbreviations

**Aβ**: Amyloid-beta; **AD**: Alzheimer’s Disease; **ATN**: Amyloid, Tau, and Neurodegeneration; **CCA**: Canonical Correlation Analysis; **CMBs**: Cerebral Microbleeds; **CSVD**: Cerebral Small Vessel Disease; **FI**: Fat Infiltration; **MCI**: Mild Cognitive Impairment; **MMSE**: MiniMental State Examination; **MRI**: Magnetic Resonance Imaging; **PACS**: Picture Archiving and Communication System; **PVSs**: Perivascular Spaces; **STRIVE**: STandards for ReportIng Vascular changes on nEuroimaging; **TMT**: Temporal Muscle Thickness; **WMHs**: White Matter Hyperintensities.
